# Supplementary material for: Biomass Derived N-Doped Porous Carbon Made from Reed Straw for an Enhanced Supercapacitor
Source: Molecules. 2023 Jun 8;28(12):4633. doi: 10.3390/molecules28124633 (PMC10301256; doi:10.3390/molecules28124633)
Supplement: Supplementary file 1 [file molecules-28-04633-s001.zip › molecules-2429649-supplementary.pdf]

Supplementary Material

Biomass Derived N-Doped Porous Carbon Made from Reed Straw for an Enhanced Supercapacitor

Yuyi Liao, Zhongtao Shang, Guangrui Ju, Dingke Wang, Qiao Yang, Yuan Wang * and Shaojun Yuan *

Low-Carbon Technology & Chemical Reaction Engineering Lab, College of Chemical Engineering, Sichuan University, Chengdu 610065, China; yimilyymail@163.com (Y.L.); taozi11281125@163.com (Z.S.); jugr0013@163.com (G.J.); dingkew0506@163.com (D.W.); yangqiaomail@163.com (Q.Y.)
* Correspondence: wangyuan2022@scu.edu.cn (Y.W.); ysj@scu.edu.cn (S.Y.)

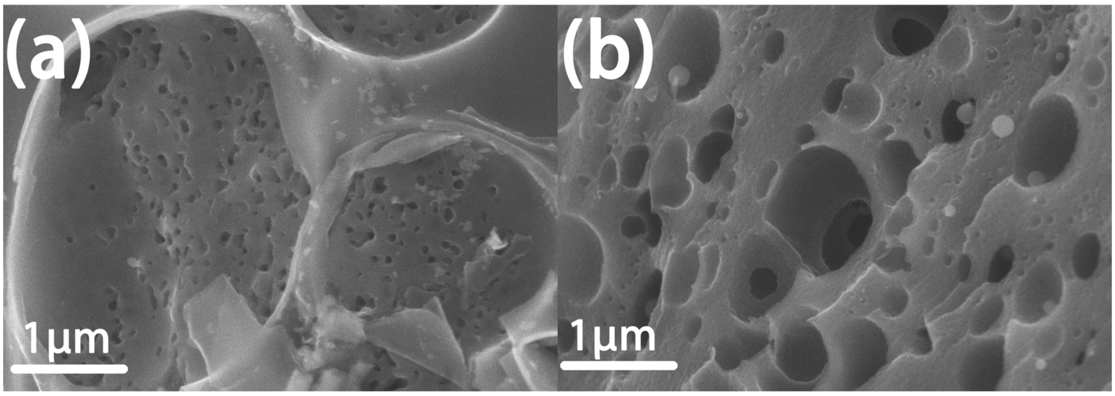

Figure S1. SEM images of (a) RSM-0.33-550 and (b) RSM-1-550.

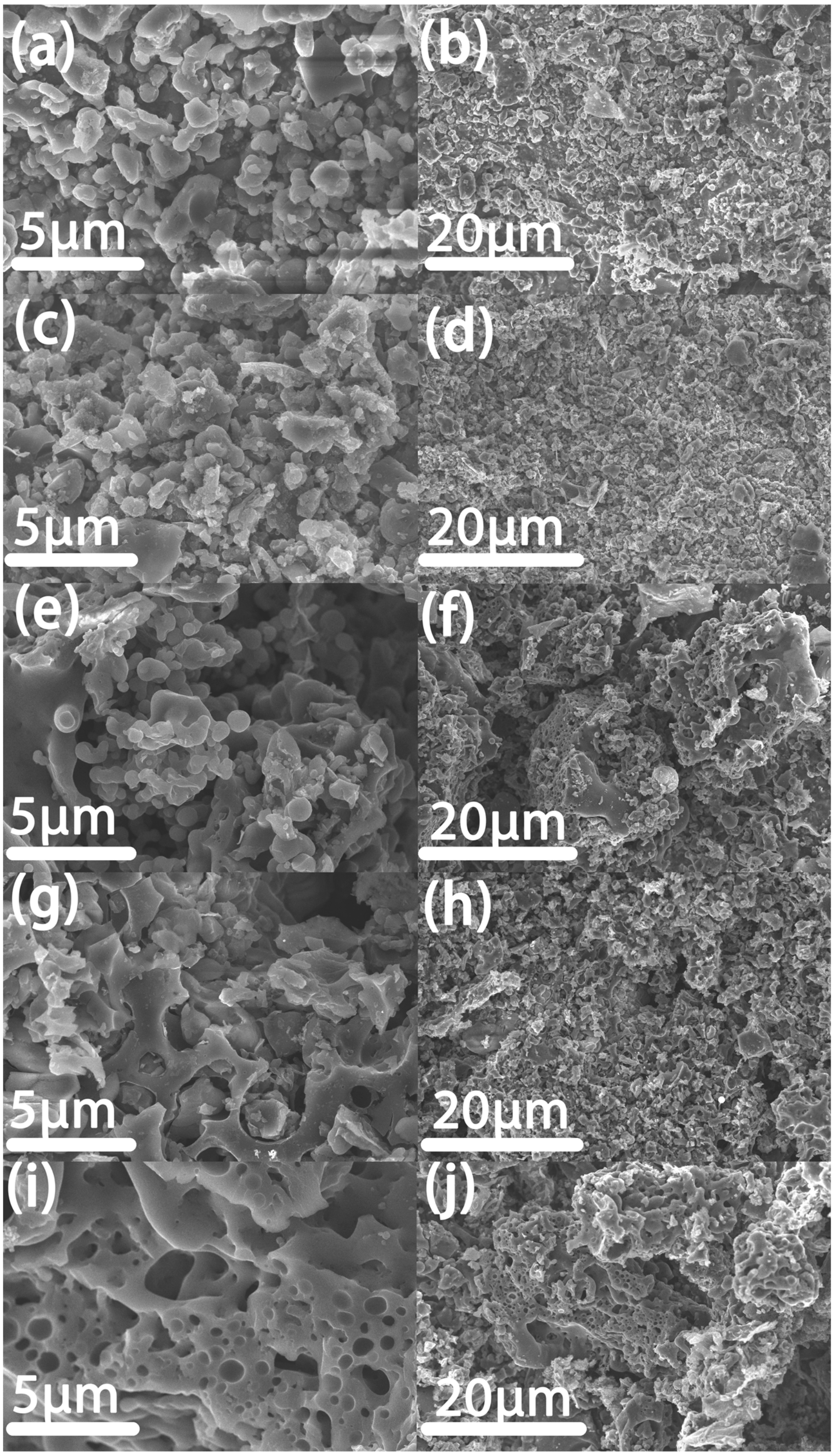

Figure S2. SEM images of (a,b) RSM-1-400, (c,d) RSM-1-500, (e,f) RSM-1-550, (g,h) RSM-1-600, and (i,j) RSM-1-650.

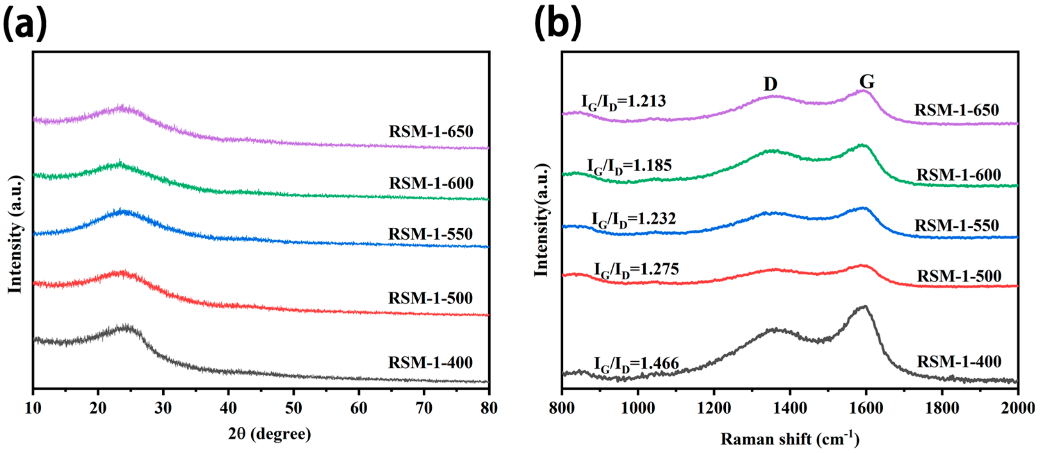

Figure S3. (a) XRD patterns of RSM-1-x (x = 400, 500, 550, 600, and 650), (b) Raman spectra of RSM-1-x (x = 400, 500, 550, 600, and 650).

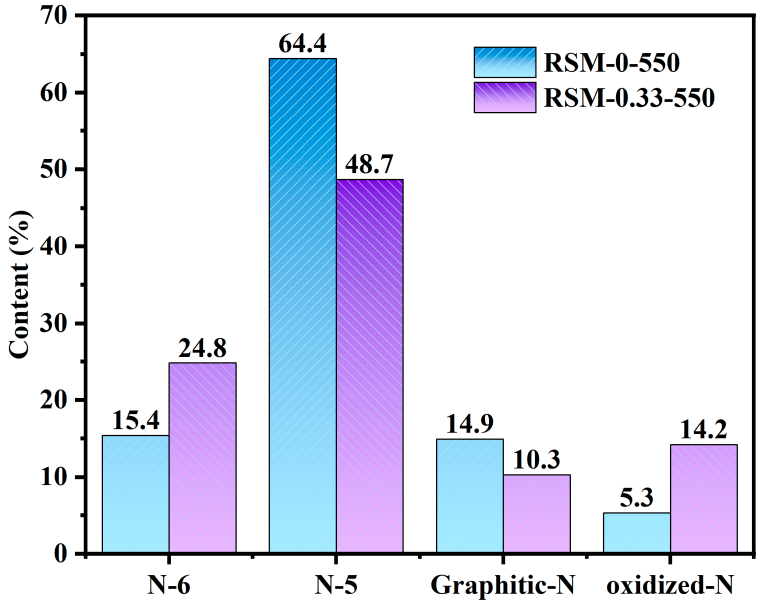

Figure S4. The content of different N species in RSM-0-550 and RSM-0.33-550.

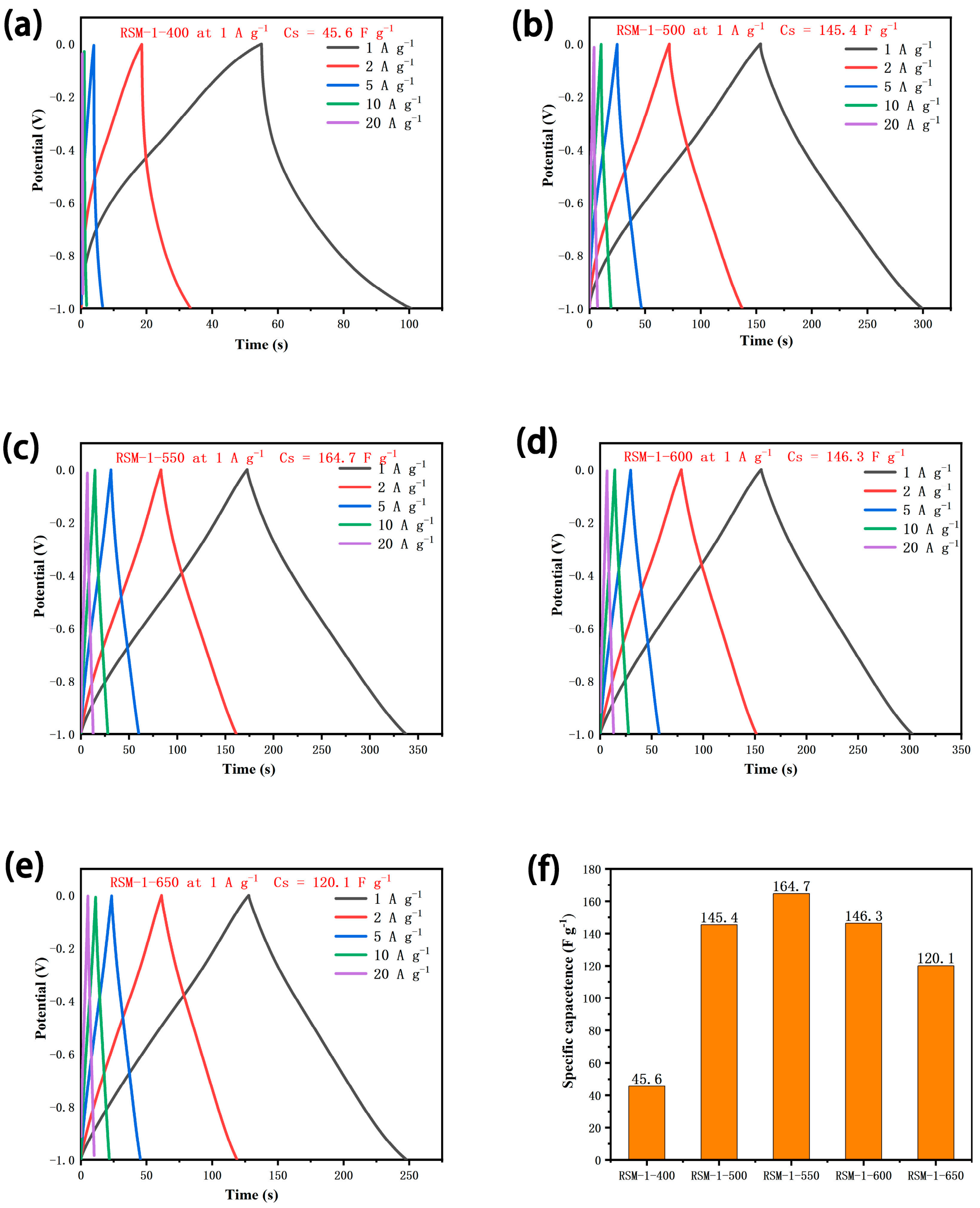

Figure S5. GCD curves of (a) RMS-1-400, (b) RMS-1-500, (c) RMS-1-550, (d) RMS-1-600, and (e) RMS-1-650 electrodes at the current densities of 1, 2, 4, 5, and 10 A g^{-1}, respectively. (f) The specific capacitance of RSM-1-X (X = 400, 500, 550, 600, and 650) at a current density of 1 A g^{-1}.

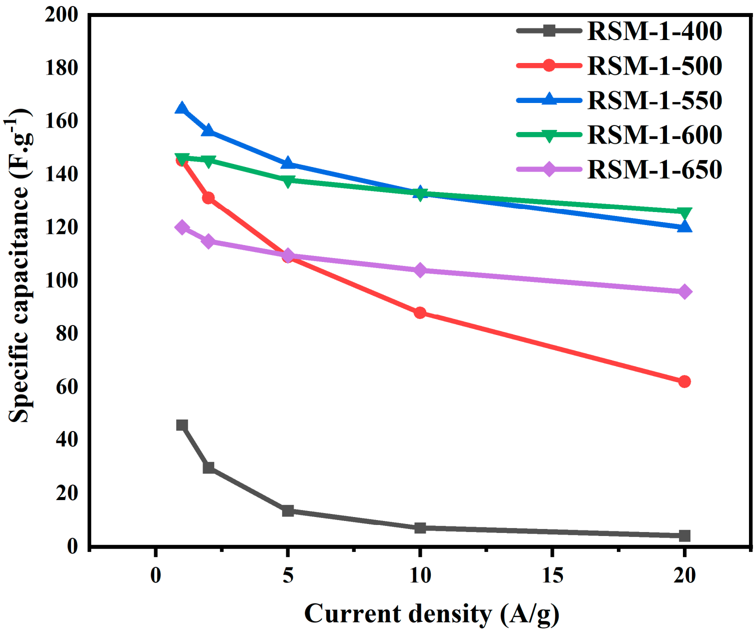

Figure S6. Rate performance of RSM-1-x (x = 400, 500, 550, 600, and 650) electrodes.

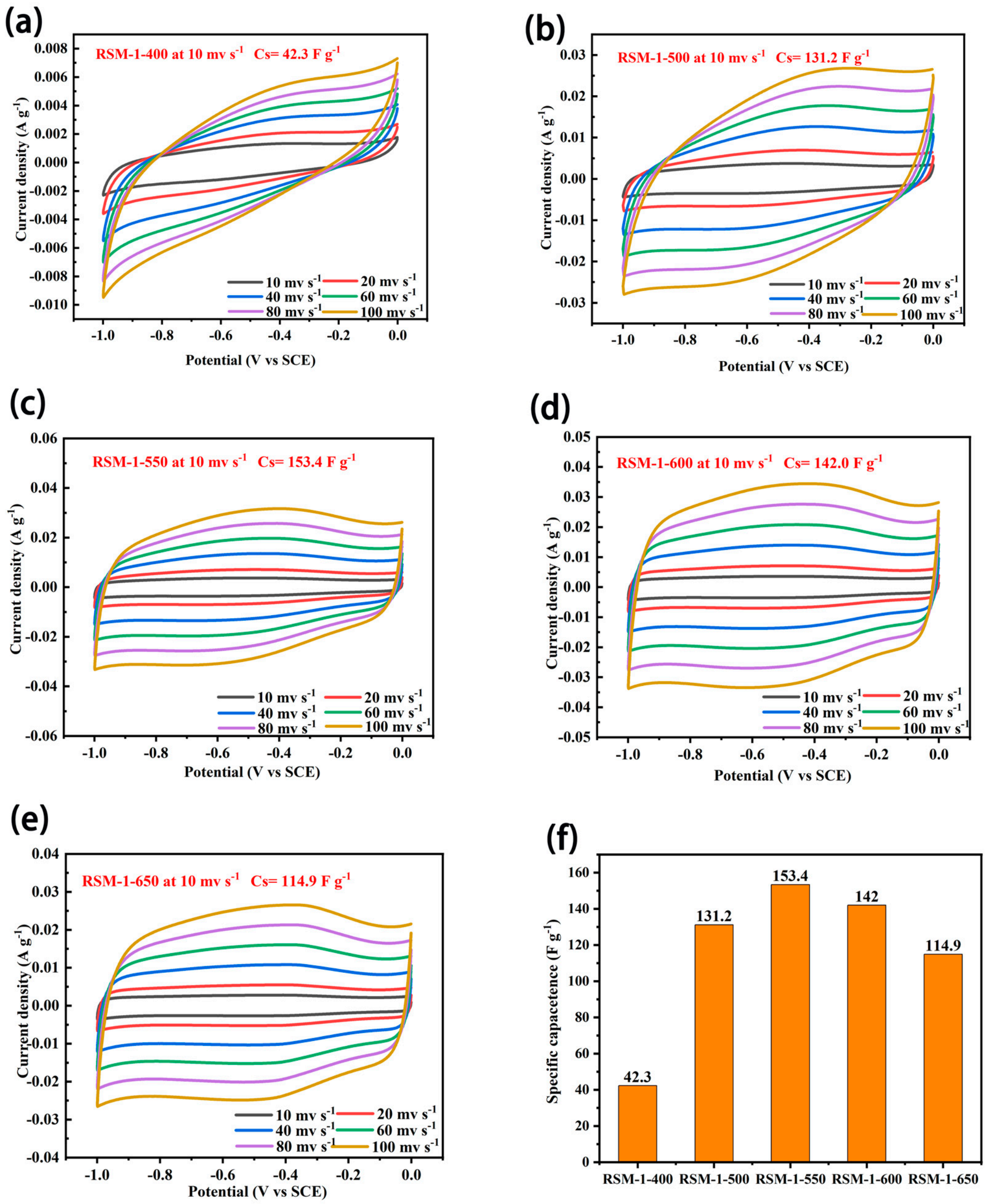

Figure S7. CV curves of (a) RSM-1-400, (b) RSM-1-500, (c) RSM-1-550, (d) RSM-1-600, and (e) RSM-1-650 electrodes at a scan rate of 10–100 mV s^{-1}. (f) The specific capacitance values of RSM-1-X (X = 400, 500, 550, 600, and 650) at a scan rate of 10 mV s^{-1}.

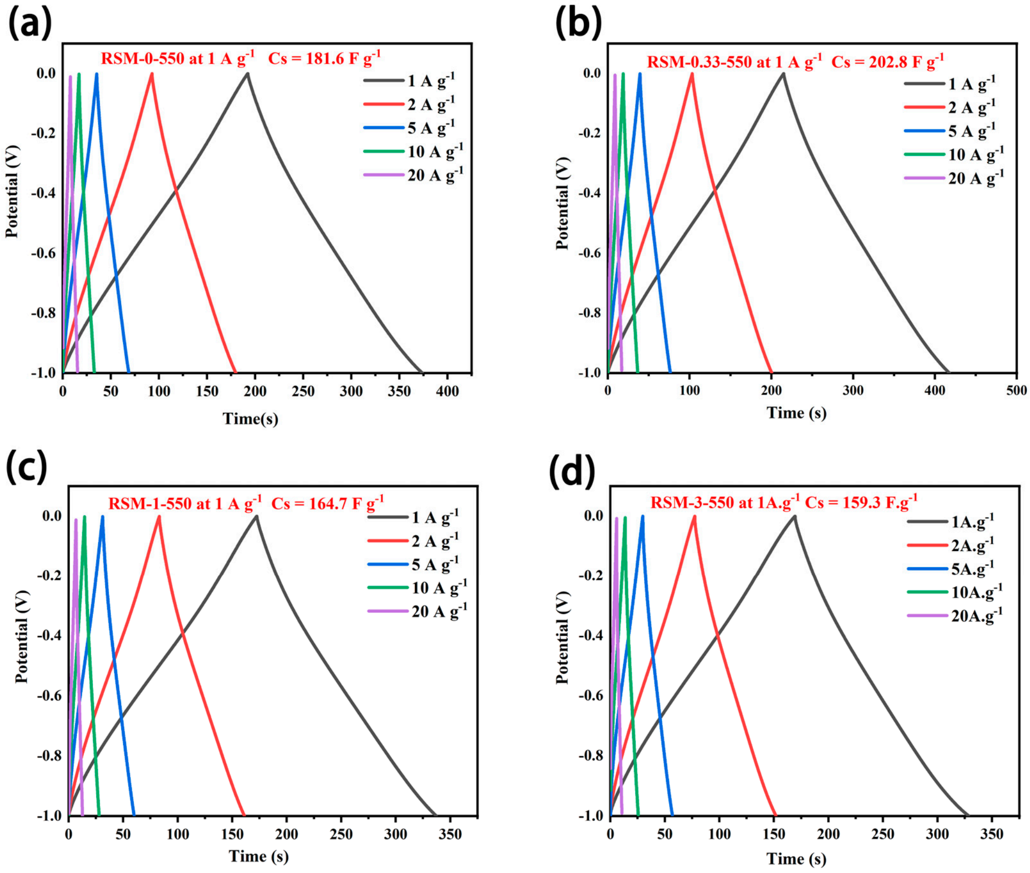

Figure S8. GCD curves of (a) RMS-0-550, (b) RMS-0.33-550, (c) RMS-1-550, and (d) RMS-3-550 electrodes at different current densities of 1, 2, 4, 5, and 10 A g^{-1}.

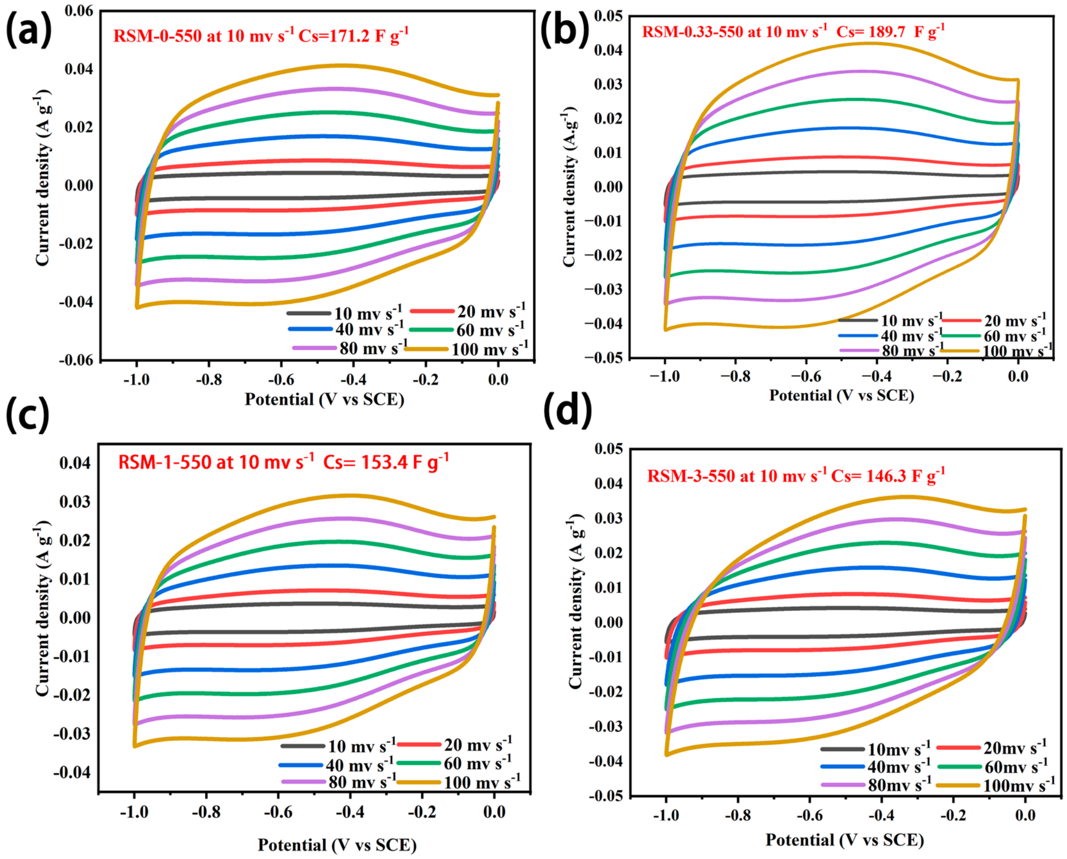

Figure S9. CV curves of (a) RMS-0-550, (b) RMS-0.33-550, (c) RMS-1-550, and (d) RMS-3-550 electrodes at a scan rate of 10–100 mV s^{-1}.

Table S1. structural parameters of RSM-0.33-550.

**Samples**	**Surface area** [a] **(m^2g^{-1})**	**Pore volume** [b] **(cm^3g^{-1})**	**Pore size** [c] **(nm)**
RSM-0.33-550	547.1	0.159	9.2
RSM-0-550	514.2	0.141	10.0

[a] BET surface area.

[b] BJH adsorption cumulative volume of pores between 17.000 and 3,000.000 Å width.

[c] BJH adsorption average pore width (4V/A).

Table S2. Electrochemical performance of RSM-0.33-550 and reported carbonaceous materials electrode in supercapacitors.

Electrode	Electrolyte	Specific capacitance ($F\ g^{-1}$)	Ref.
**RMS-0.33-550**	**6 M KOH**	**202.8 (1 A g^{-1})**	**This work**
APG-1%	6 M KOH	158.5 (1 A g^{-1})	Journal of Power Sources,2020,472,228610.
Micro-, meso- and macroporous	1 M $(C_2H_5)_4N(BF_4)$	157 (0.5 mA cm^{-2})	Sci. Rep. 2016, 6, 21182.
Exfoliated carbon nanotubes	3 M NaOH	154.4 (1 A g^{-1})	J. Power Sources 2011, 196, 5209–5214.
P doping Graphene	6 M KOH	115 (0.05 A g^{-1})	Chem. –Eur. J. 2015, 21, 80–85.
B, N doping Graphene	1 M H_2SO_4	29.7 (0.1 A g^{-1})	J. Mater. Chem. A 2014, 2, 9532–9540.
mesopores carbon	1 M H_2SO_4	118 (5 mV s^{-1})	Chem. Int. Ed. 2017, 56, 5454–5459.
multi wall carbon nanotube	6 M KOH	179 (0.5 A g^{-1})	J. Renew. Sustain. Ener. 2016, 8, 014101.
N-functionalized graphene	6 M KOH	100 (0.1 V s^{-1})	ACS Appl. Mater. Interfaces 2017, 9, 710–721.
N-containing graphene	0.1 M KOH	50 (0.7 A g^{-1})	Appl. Mat. Today 2017, 9, 204–211.
boron-doped graphene	6 M KOH	50 (0.1 A g^{-1})	ChemSusChem 2014, 7, 1102–1106.
Hierarchical porous carbon	0.5M H_2SO_4	115	Electrochim. Acta 2013, 113, 481–489.
Core-shell composite/MnO2	1 M Na_2SO_4	101 (0.05 A g^{-1})	RSC Adv. 2016, 6, 64811–64817.
Few layered graphene	Aq. H_2SO_4	151	Arab. J. Chem. 2017, 10, 556–565.
N, O, and P decorated carbon	6 M KOH	206	Electrochim. Acta 2015, 176 982–988.

Activated carbon	1.5 M Na_2SO_4	82.5 (50 mV s^{-1})	J. New Mater. Electron. Syst. 2015, 18, 1–9.
